# Electrostatic interactions mediate the nucleation and growth of a bacterial functional amyloid

**DOI:** 10.3389/fmolb.2023.1070521

**Published:** 2023-01-12

**Authors:** Sujeet S. Bhoite, Divya Kolli, Mark A. Gomulinski, Matthew R. Chapman

**Affiliations:** Department of Molecular, Cellular and Developmental Biology, University of Michigan, Ann Arbor, MI, United States

**Keywords:** CsgA, curli, gatekeeper, amyloid formation, functional amyloid, biofilm

## Abstract

Bacterial biofilm formation can have severe impacts on human and environmental health. Enteric bacteria produce functional amyloid fibers called curli that aid in biofilm formation and host colonization. CsgA is the major proteinaceous component of curli amyloid fibers and is conserved in many gram-negative enteric bacteria. The CsgA amyloid core consists of five imperfect repeats (R1-R5). R2, R3, and R4 have aspartic acid (D) and glycine (G) residues that serve as “gatekeeper” residues by modulating the intrinsic aggregation propensity of CsgA. Here, using mutagenesis, salt-mediated charge screening, and by varying pH conditions, we show that the ability of CsgA variants to nucleate and form amyloid fibers is dictated by the charge state of the gatekeeper residues. We report that in *Citrobacter youngae* CsgA, certain arginine (R) and lysine (K) residues also act as gatekeeper residues. A mechanism of gatekeeping is proposed wherein R and K residues electrostatically interact with negatively charged D residues, tempering CsgA fiber formation.

## Introduction

Bacteria can be found in biofilms, complex communities enclosed within an extracellular matrix (ECM) (Costerton et al., 1995; Costerton, 1999). The ECM confers resistance to the underlying cells from physical and chemical stressors such as dehydration, predation, and antibiotics among others (Elasri Mohamed and Miller Robert, 1999; Daniel and O’Toole George, 2005; Roberts and Stewart, 2005; Matz et al., 2008; Mulcahy et al., 2008; Truelstrup Hansen and Vogel, 2011; Haaber et al., 2012; Kostakioti et al., 2013). Biofilms have been extensively studied due to their impact on human health and disease (Costerton, 1999; Donlan and William Costerton 2002; Parsek and Singh, 2003; Motta et al., 2021). The physiological and biochemical basis of biofilm formation by enteric bacteria is of particular interest due to connections with host digestion, immunity, and pathologies (Banwell et al., 1985; Macfarlane et al., 1997; Palestrant et al., 2004; de Vos, 2015; Donaldson et al., 2016). The *Enterobacteriaceae* family includes many species which colonize the human gut and are implicated in disease, such as *Salmonella enterica*, *Serratia marcescens*, *Klebsiella*, *Yersinia pestis*, and *Escherichia coli* (*E. coli*) (Hufnagel David et al., 2015). The biofilms produced by enteric bacteria are composed of functional amyloids, polysaccharides, and extracellular DNA (eDNA) (Steinberger and Holden, 2005; Jonas et al., 2007; Qin et al., 2007; Izano Era et al., 2008; Guiton Pascale et al., 2009; Kostakioti et al., 2013). Of interest is the functional amyloid curli, produced by certain enteric bacteria and first identified in *E. coli* (Chapman Matthew et al., 2002). Curli amyloid fibrils have been shown to be important in biofilm formation by mediating initial surface attachment and are an important structural component of the overall biofilm architecture (Kikuchi et al., 2005; Hufnagel et al., 2013; Hung et al., 2013).

Two divergently transcribed operons containing seven genes control the expression, secretion, and formation of the curli fibril (Chapman Matthew et al., 2002). The major curli subunit is an aggregation-prone protein called CsgA. CsgA is predicted to be mostly intrinsically disordered until its polymerization is initiated in the extracellular space upon encountering the surface-anchored nucleator protein, CsgB (Sujeet et al., 2019). Once polymerized, the resulting curli amyloid fibrils provide structural integrity to the biofilm. CsgA has been predominantly studied in *E. coli* cells. *E. coli* lacking CsgA are deficient in biofilm formation and surface attachment (Chapman Matthew et al., 2002; Tursi and Tükel, 2018). Interestingly, CsgA homologs from diverse bacterial species have been shown to complement *E. coli* CsgA deletion *in vivo* and fragments of CsgA homolog fibers can seed the amyloidogenic aggregation of *E. coli* CsgA *in vitro* (Zhou et al., 2012). Since most bacterial communities are composed of multiple species, it is plausible that CsgA homologs from different species can be shared to build biofilms containing a heterogeneous matrix and population. This process is of particular interest for the diverse range of amyloid-producing enteric bacteria in the human gut and introduces the importance of studying CsgA homologs in various enteric bacterial species. Given the importance of CsgA in biofilm formation, various *in vivo* and *in vitro* methods have been developed to study how CsgA aggregates into amyloid fibers (Zhou et al., 2013).

*In vitro* Thioflavin-T (ThT) fluorescence studies have shown that the polymerization of *E. coli* CsgA follows a nucleation dependent polymerization (NDP) model (Jain et al., 2017). The sigmoidal aggregation pattern includes the initial nucleation lag phase followed by rapid fiber polymerization and a final stationary phase (Chiti and Dobson, 2017; Jain et al., 2017). CsgA is comprised of five conserved imperfect repeat units designated as R1-R5 (Wang et al., 2007). Each repeat unit is predicted to form a β-helix-like structure with a characteristic strand-loop-strand motif (DeBenedictis et al., 2017). The Q-X4-N-X5-Q consensus sequence of the repeat units is important for initiating and propagating CsgA polymerization *via* side chain interactions of the glutamine (Q) and asparagine (N) residues (Wang and Chapman, 2008). Interestingly, units R2, R3 and R4 contain certain aspartic acid (D) and glycine (G) residues, termed as “gatekeepers,” which impede the intrinsic aggregation propensity of *E. coli* CsgA, a phenomenon termed as “gatekeeping” (Wang et al., 2010). Substitution of the gatekeeper residues with corresponding residues in R1 and R5 repeat units lead to increased aggregation propensity of CsgA and a significant decrease in the lag phase, indicating that gatekeepers may play an important role in modulating CsgA nucleation (Wang et al., 2010). Charged amino acid residues like lysine (K), arginine (R), glutamic acid (E), and aspartic acid (D) have been shown to serve as gatekeeper residues by interfering with β-sheet formation due to large and flexible sidechains (e.g., R, K) or charge-charge repulsion (Reumers et al., 2009; Beerten et al., 2012). It has also been shown that electrostatic interaction between positively and negatively charged amino acid residues can modulate amyloidogenesis in proteins (Yun et al., 2007; Meisl et al., 2017; Lin et al., 2020). We previously found that wild-type *Citrobacter youngae* CsgA (CY CsgA) does not contain the same gatekeeper D residues as *E. coli* CsgA and that CY CsgA polymerizes very quickly compared to other CsgA homologs (Bhoite et al., 2022). In addition, a mutated CY CsgA variant where the *E. coli* gatekeeper (^GK^) D residues were added, named CY^GK^ CsgA (CY CsgA^V78D/S89D/N125D^), displayed slower polymerization rates than CY CsgA (Bhoite et al., 2022). Here, we investigated the role of gatekeeper residues in modulating amyloidogenesis of the CY^GK^ CsgA mutant. We hypothesized that the introduction of gatekeeper D residues in CY CsgA could either control polymerization by a) repulsion between the negatively charged D residues preventing compact amyloid formation or b) intramolecular interactions with D residues could be stabilizing CsgA monomers delaying the formation of a polymerization competent species.

Sequence alignment of CsgA homologs from diverse species revealed that a) not all CsgA homologs have the conserved D gatekeeper residues and b) in CsgA homologs with conserved D residues, two positively charged residues, namely arginine (R) and lysine (K), are highly conserved (Supplementary Figure S1). We thus hypothesized that the gatekeeping ability is in part conferred by the negatively charged D residues and the positively charged R and K residues electrostatically interacting with each other, modulating the formation of the aggregation prone β-helix conformation of CsgA. Wild-type CY CsgA contains the positively charged R62 and K107 residues but lacks most of the gatekeeper D residues (Supplementary Figure S1). The introduction of gatekeeper D residues could allow for electrostatic interactions with the native R and K residues to form, leading to the increase in lag phase observed in the CY^GK^ CsgA mutant.

In this study, CY^GK^ CsgA was used to investigate the mechanism behind gatekeeping activity. We report that pH-induced charge manipulation and salt-mediated charge screening significantly impacted gatekeeping activity in CY^GK^ CsgA. We also identify new positively-charged gatekeeper residues, R62 and K107, and show that deletion or substitution of these residues negatively impacted gatekeeping activity. We demonstrate that charge neutralization and mutation of the positively charged residues as well as partial loss of negative charge on the D78, D89, and D125 residues abolished gatekeeping activity. Based on our data, we propose a mechanism wherein an electrostatic interaction, namely R or K residues interacting with D residues, represents one mechanism of gatekeeping CY^GK^ CsgA nucleation.

## Results

### Substitution of positively charged R and K residues leads to increased nucleation rates

**FIGURE 1:**
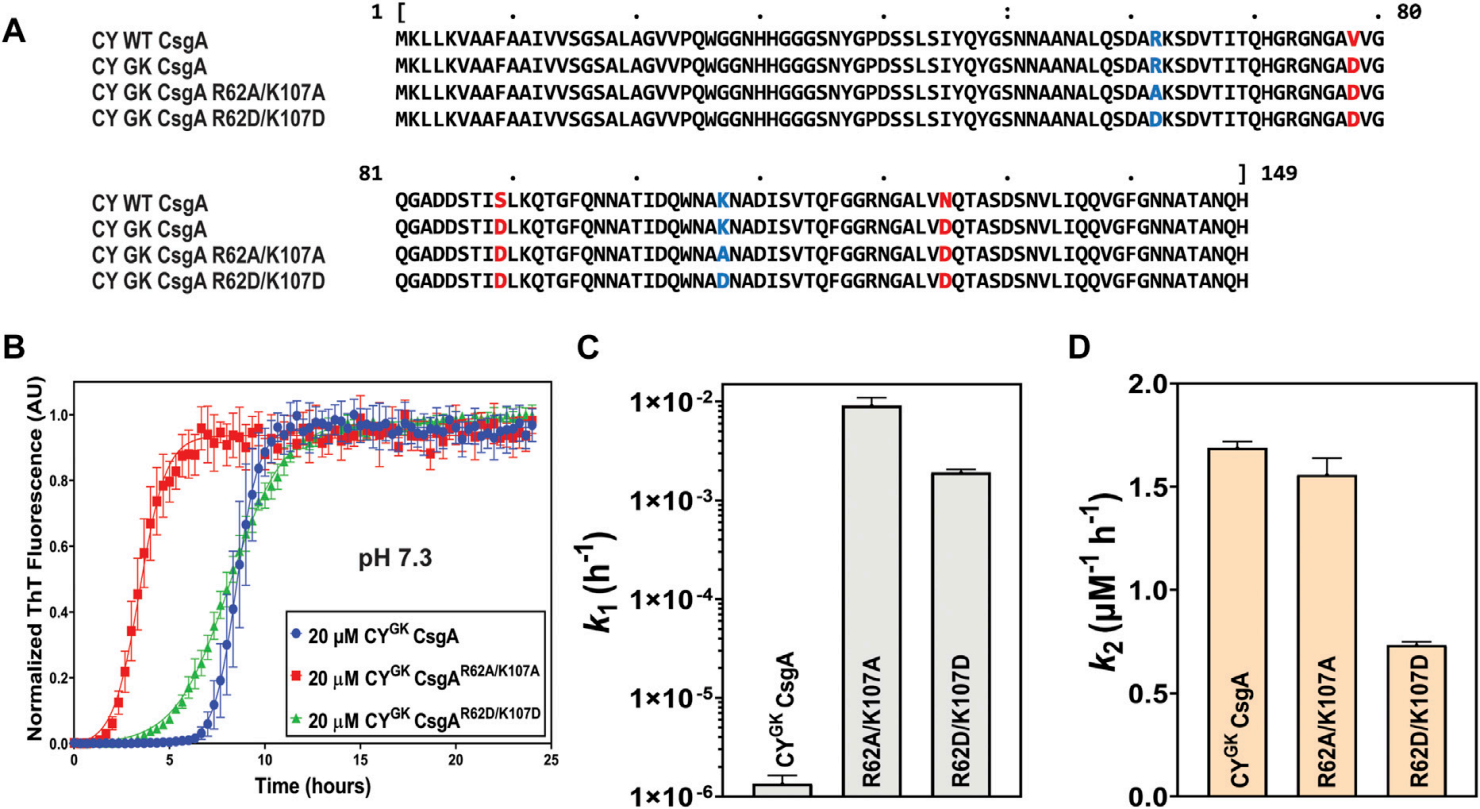
Gatekeeping function is affected by R and K residues. Analysis of aggregation kinetics of CY^GK^ CsgA, CY^GK^ CsgA^R62A/K107A^, and CY^GK^ CsgA^R62D/K107D^ at 37°C, pH 7.3. **(A)** Sequence alignment of wild-type CY CsgA, CY^GK^ CsgA, CY^GK^ CsgA^R62A/K107A^, and CY^GK^ CsgA^R62D/K107D^ with aspartic acid gatekeeper residues in red and mutated arginine and lysine in blue. Alignment was created using MView (https://www.ebi.ac.uk/Tools/msa/mview/). **(B)** Thioflavin T (ThT) fluorescence assay using 20 μM of CY^GK^ CsgA, CY^GK^ CsgA^R62A/K107A^, and CY^GK^ CsgA^R62D/K107D^. **(C)** Nucleation rates *k*_*1*_ of amyloidosis on a log scale and **(D)** Growth rates *k*_*2*_ of amyloid fiber propagation on a linear scale. [Error bars represent Standard Error of Mean (SEM) for ThT assay and Standard Deviation (SD) for k_1_ and k_2_ of three replicates].

Sequence alignments revealed the presence of conserved positively charged R and K residues in diverse CsgA homologs (Supplementary Figure S1). We hypothesized that the positively charged R62 and K107 residues play an important role in gatekeeping CY^GK^ CsgA polymerization in addition to the earlier discovered gatekeeper D78, D89, and D125 residues. To test this hypothesis, the positively charged R62 and K107 residues in CY^GK^ CsgA (CY CsgA^V78D/S89D/N125D^) were mutated either to a neutral alanine (CY^GK^ CsgA^R62A/K107A^) or a negatively charged aspartic acid (CY^GK^ CsgA^R62D/K107D^) (Figure 1A). We purified these two mutants along with CY^GK^ CsgA and studied their aggregation kinetics *in vitro* using Thioflavin-T (ThT) fluorescence assays (Naiki et al., 1989; Xue et al., 2017). As previously reported (Bhoite et al., 2022), CY^GK^ CsgA displayed a lag phase of ∼6 to 7 h at pH 7.3 (Figure 1B). Interestingly, at pH of 7.3, CY^GK^ CsgA^R62A/K107A^ and CY^GK^ CsgA^R62D/K107D^ displayed a reduced lag phase of ∼1 h and ∼3 h, respectively, compared to CY^GK^ (Figure 1B). The aggregation kinetic curves were fitted by the Finke-Watzky two-step model of nucleation and growth (Morris et al., 2008). This model allowed estimation of the nucleation rate constant *k*_*1*_ and autocatalytic growth constant *k*_*2*_ for CY^GK^ CsgA polymerization (Supplementary Figure S2). The nucleation rate constant *k*_*1*_ of CY^GK^ CsgA^R62A/K107A^ and CY^GK^ CsgA^R62D/K107D^ showed a four log increase in the nucleation rate compared to CY^GK^ CsgA (Figure 1C). This significant increase in the nucleation rate indicated that R62 and K107 residues are important for nucleus formation and may function as gatekeeper residues. The growth rate constant *k*_*2*_ did not change between CY^GK^ CsgA and CY^GK^ CsgA^R62A/K107A^, but CY^GK^ CsgA^R62D/K107D^ showed a two times slower growth rate suggesting there may be negative charge repulsion impacting CsgA polymerization at the growth phase (Figure 1D).

### Salt-mediated charge-masking negatively affects the gatekeeping function of R, K, and D residues

**FIGURE 2:**
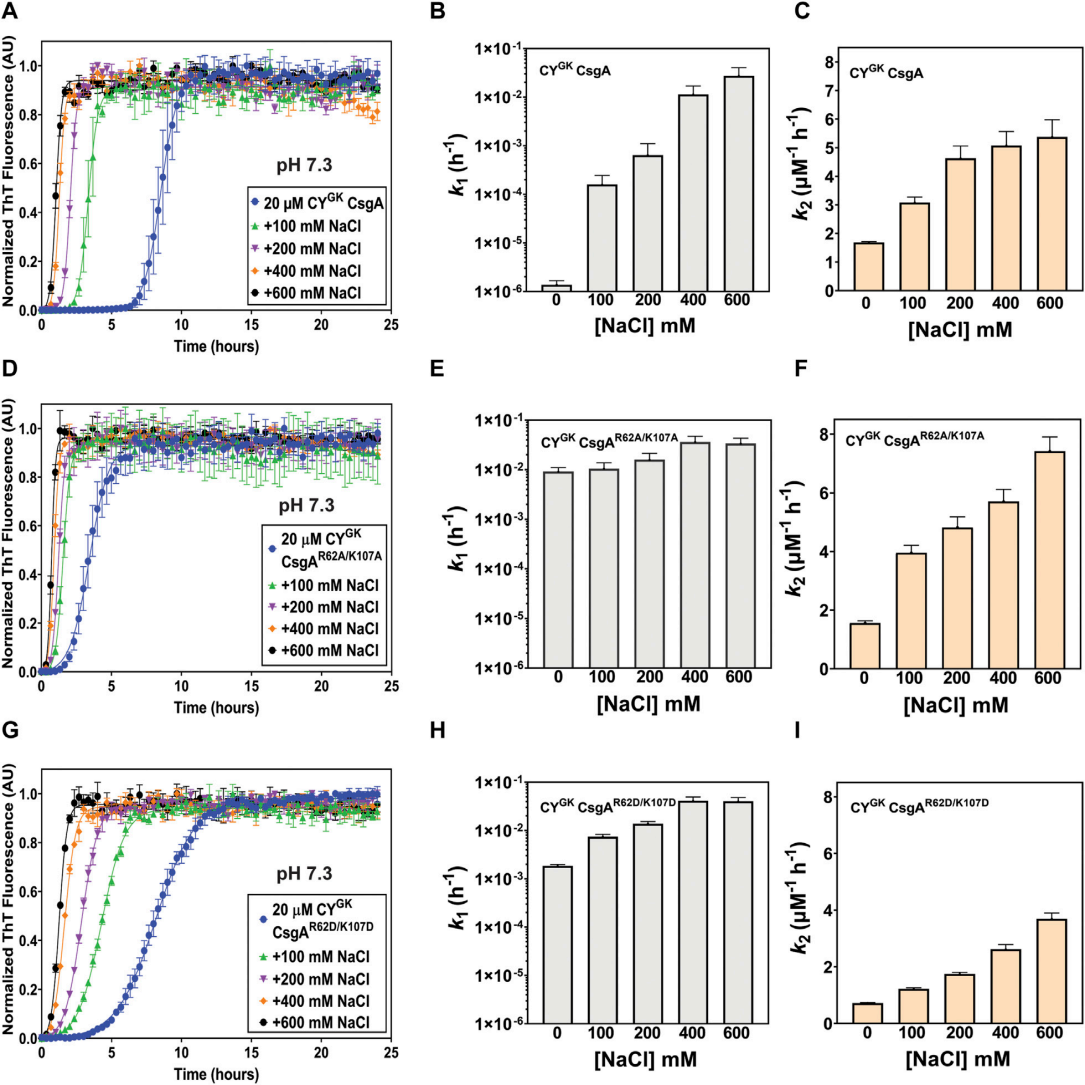
Gatekeeping function is sensitive to salt-mediated charge screening. Analysis of aggregation kinetics of CY^GK^ CsgA, CY^GK^ CsgA^R62A/K107A^, and CY^GK^ CsgA^R62D/K107D^ at 37°C, pH 7.3 in presence of increasing NaCl concentrations. **(A)** ThT fluorescence assay of CY^GK^ CsgA. **(B)** Nucleation rates *k*_*1*_ of amyloidosis (log scale) of CY^GK^ CsgA. **(C)** Growth rates *k*_*2*_ of amyloid fiber propagation (linear scale) of CY^GK^ CsgA. **(D)** ThT fluorescence assay of CY^GK^ CsgA^R62A/K107A^. **(E)** Nucleation rates *k*_*1*_ of amyloidosis (log scale) of CY^GK^ CsgA^R62A/K107A^. **(F)** Growth rates *k*_*2*_ of amyloid fiber propagation (linear scale) of CY^GK^ CsgA^R62A/K107A^. **(G)** ThT fluorescence assay of CY^GK^ CsgA^R62D/K107D^. **(H)** Nucleation rates *k*_*1*_ of amyloidosis (log scale) of CY^GK^ CsgA^R62D/K107D^ and **(I)** Growth rates *k*_*2*_ of amyloid fiber propagation (linear scale) of CY^GK^ CsgA^R62D/K107D^. (Error bars represent SEM for ThT assay and SD for *k*_*1*_ and *k*_*2*_ of three replicates).

Electrostatic interactions between the negatively charged D78, D89, and D125 residues and the positively charged R62 and K107 gatekeeper residues in CsgA might play a role in slowing down the nucleation rate of CY^GK^ CsgA. Aggregation reactions were carried out at varying concentrations of NaCl. Increasing NaCl concentrations was predicted to increase nucleation rates as the charge screening would disrupt electrostatically-mediated gatekeeping. In CY^GK^ CsgA we observed that with increasing salt concentrations, the lag phase decreased with a concomitant increase in the nucleation rates and a four log increase in the nucleation rate at the highest salt concentration of 600 mM (Figures 2A, B). Conversely, the salt-mediated charge screening had less of an effect on the lag phase and nucleation rates of CY^GK^ CsgA^R62A/K107A^ and CY^GK^ CsgA^R62D/K107D^, which both would lack the electrostatic interactions between a positively charged gatekeeper residue and a D gatekeeper residue. The nucleation rates of CY^GK^ CsgA^R62A/K107A^ and CY^GK^ CsgA^R62D/K107D^ between 0 mM and 600 mM salt increased only by .5 and 1.3 log respectively (Figures 2D, E, G, H). The growth rates of CY^GK^ CsgA, CY^GK^ CsgA^R62A/K107A^, and CY^GK^ CsgA^R62D/K107D^ did not increase significantly (Figures 2C, F, I).

### Negative charge on D residues is necessary for gatekeeping function

**FIGURE 3:**
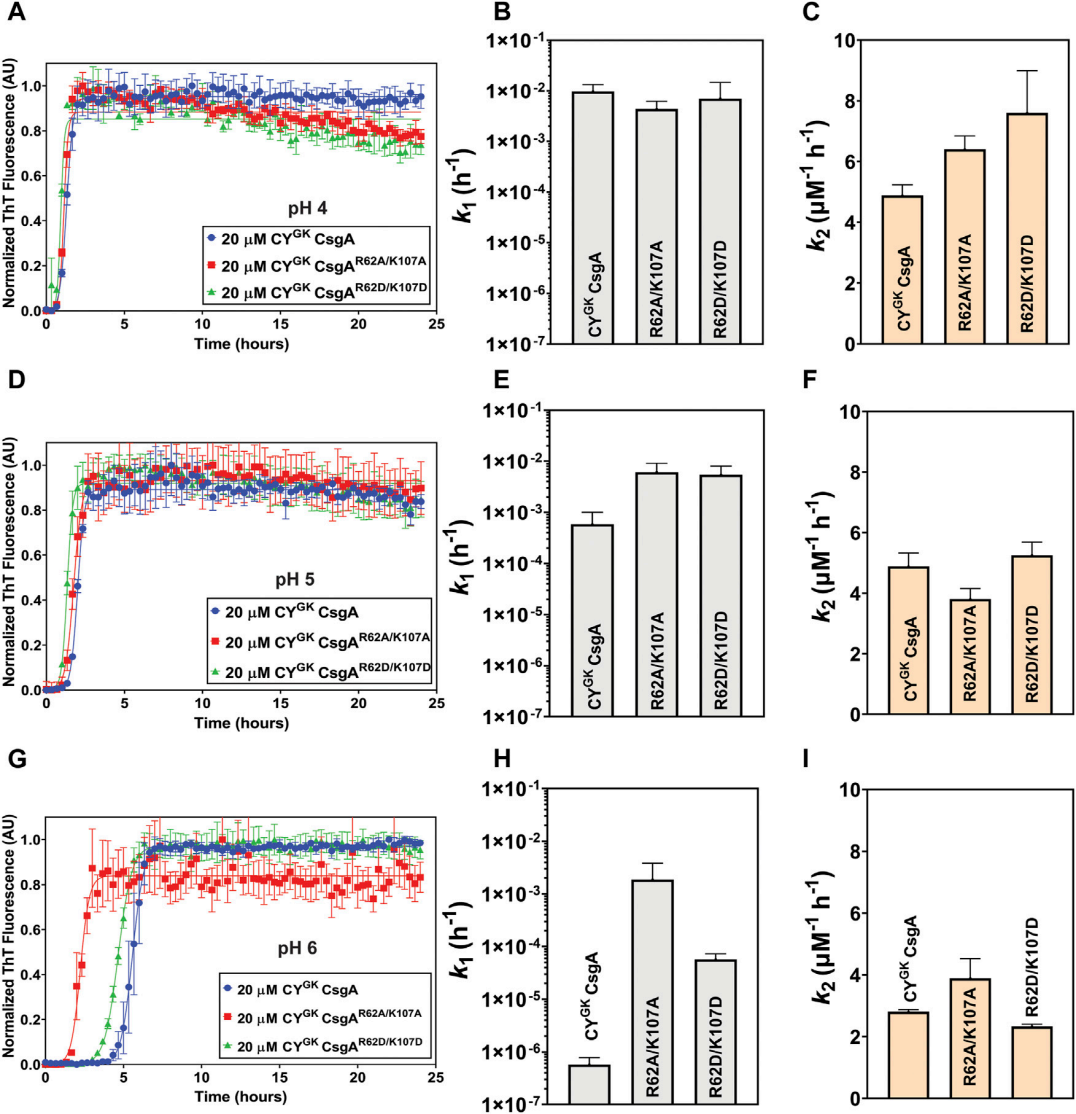
pH-induced charge neutralization of D residues negatively affects gatekeeping function. Analysis of aggregation kinetics of CY^GK^ CsgA, CY^GK^ CsgA^R62A/K107A^, and CY^GK^ CsgA^R62D/K107D^ at 37°C, pH 4. **(A)** ThT fluorescence assay. **(B)** Nucleation rates *k*_*1*_ of amyloidosis (log scale) and **(C)** Growth rates *k*_*2*_ of amyloid fiber propagation (linear scale). Analysis of aggregation kinetics of CY^GK^ CsgA, CY^GK^ CsgA^R62A/K107A^, and CY^GK^ CsgA^R62D/K107D^ at 37°C, pH 5. **(D)** ThT fluorescence assay. **(E)** Nucleation rates *k*_*1*_ of amyloidosis (log scale) and **(F)** Growth rates *k*_*2*_ of amyloid fiber propagation (linear scale). Analysis of aggregation kinetics of CY^GK^ CsgA, CY^GK^ CsgA^R62A/K107A^, and CY^GK^ CsgA^R62D/K107D^ at 37°C, pH 6. **(G)** ThT fluorescence assay. **(H)** Nucleation rates *k*_*1*_ of amyloidosis (log scale) and **(I)** Growth rates *k*_*2*_ of amyloid fiber propagation (linear scale). (Error bars represent SEM for ThT assay and SD for *k*_*1*_ and *k*_*2*_ of three replicates).

The degree of ionization and hence the charge states of an amino acid can change depending on the pH of the buffer solution. The Protparam Tool from Expasy calculated the theoretical pI of CY^GK^ CsgA to be 5.6. We studied the aggregation kinetics of the three proteins at pH 4 and pH 5 to test the effect of charged states of R, K, and D residues on gatekeeping activity. The theoretical p*K*_a_ of the ionizable carboxylic acid group in D residues is 3.9. However, in the context of the entire protein, the p*K*_a_ values differ significantly compared to the theoretical values, especially in intrinsically disordered proteins (Thurlkill et al., 2006; Quijada et al., 2007; Grimsley et al., 2009; Pahari et al., 2019). Noteworthy, is the observation that the experimentally calculated p*K*_a_ values of D residues can range from .5 to 9.9 (Pahari et al., 2019). We thus reasoned that the p*K*_a_ of D78, D89, and D125 residues in CsgA could be different from the theoretical value but that the balance of ionization states of the aspartic acids will trend towards protonation at lower pH conditions and towards deprotonation as the pH is increased. We hypothesized that in CY^GK^ CsgA at pH 4 the D78, D89, and D125 gatekeeper residues would trend towards protonation leading to a loss of electrostatic interactions with positively charged R62 and K107 residues. As the pH increases to 5, the equilibrium of the charge state of the D78, D89, and D125 gatekeeper residues would shift more towards deprotonation leading to increased interaction and, therefore, increased gatekeeping function. CY^GK^ CsgA, CY^GK^ CsgA^R62A/K107A^ and CY^GK^ CsgA^R62D/K107D^ at pH 4 displayed similar aggregation kinetics with significantly increased nucleation and growth rates compared to those at pH 7.3 (compare Figures 1B–D, 3A–C). Moreover, there was less than 1 log difference in the nucleation rates and less than 1.4 times difference in the growth rates between CY^GK^ CsgA, CY^GK^ CsgA^R62A/K107A^, and CY^GK^ CsgA^R62D/K107D^ at pH 4 (Figures 3B, C). As the pH was increased to pH 5, we observed an increase in the lag phase of CY^GK^ CsgA compared to pH 4 (Figures 3D–F). The nucleation rate of CY^GK^ CsgA at pH 5 was 1.2 log lower than that at pH 4 while the nucleation rates of CY^GK^ CsgA^R62A/K107A^ and CY^GK^ CsgA^R62D/K107D^ at pH 5 did not significantly change from pH 4 (Figure 3E). As the pH increased from pH 4 to pH 5, it would be predicted that D residues might deprotonate and become negatively charged. Thus, at pH 5 in CY^GK^ CsgA we observed increased lag phase of aggregation and significantly decreased nucleation rates compared to pH 4, indicating that gatekeeping function at the nucleation level depends in part on the charge state of D78, D89, and D125 gatekeeper residues.

**FIGURE 4:**
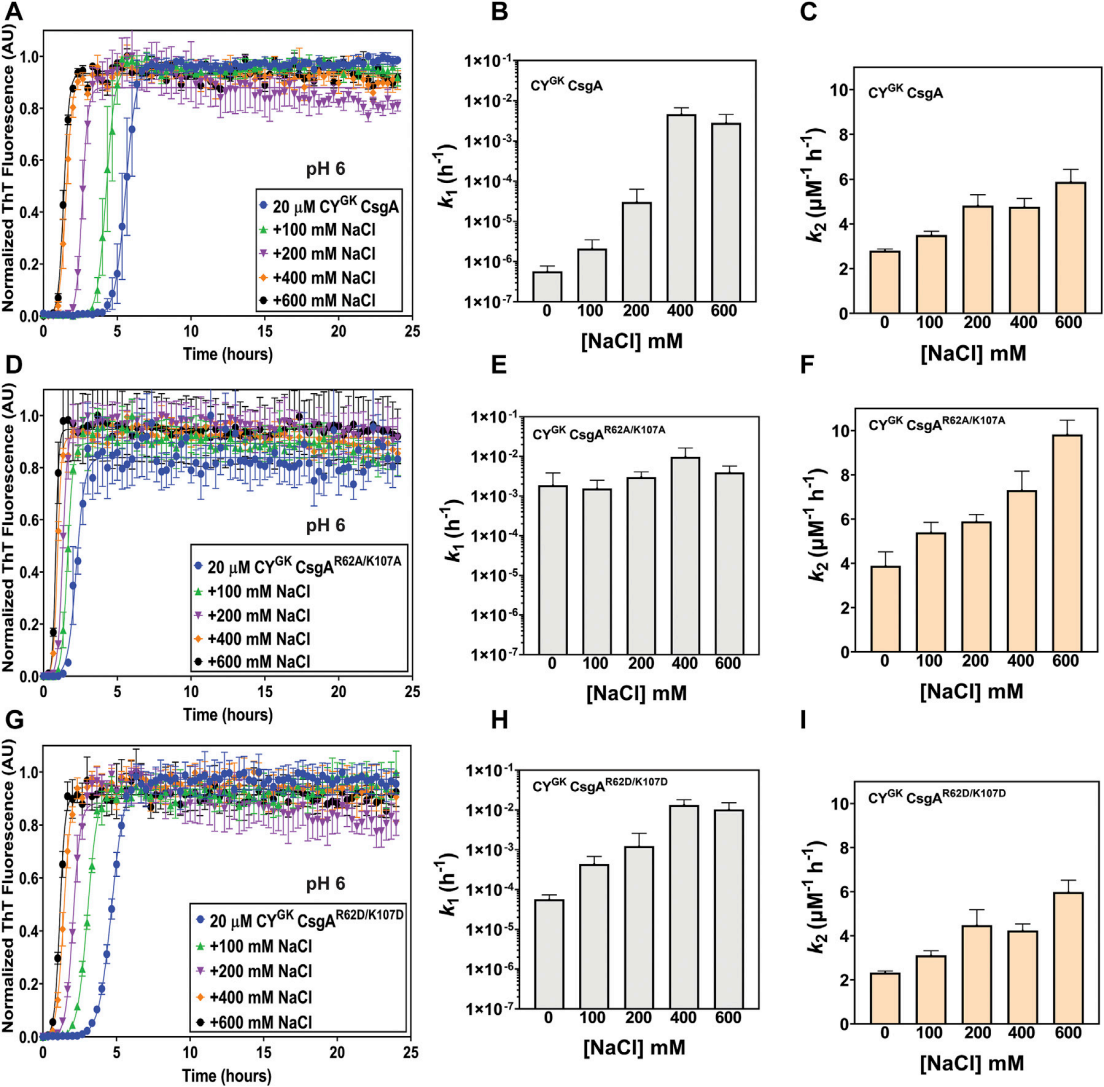
Salt-mediated charge screening reverses gatekeeping function. Analysis of aggregation kinetics of CY^GK^ CsgA, CY^GK^ CsgA^R62A/K107A^, and CY^GK^ CsgA^R62D/K107D^ at 37°C, pH 6 in presence of increasing NaCl concentrations. **(A)** ThT fluorescence assay of CY^GK^ CsgA. **(B)** Nucleation rates *k*_*1*_ of amyloidosis (log scale) of CY^GK^ CsgA. **(C)** Growth rates *k*_*2*_ of amyloid fiber propagation (linear scale) of CY^GK^ CsgA. **(D)** ThT fluorescence assay of CY^GK^ CsgA^R62A/K107A^. **(E)** Nucleation rates *k*_*1*_ of amyloidosis (log scale) of CY^GK^ CsgA^R62A/K107A^. **(F)** Growth rates *k*_*2*_ of amyloid fiber propagation (linear scale) of CY^GK^ CsgA^R62A/K107A^. **(G)** ThT fluorescence assay of CY^GK^ CsgA^R62D/K107D^. **(H)** Nucleation rates *k*_*1*_ of amyloidosis (log scale) of CY^GK^ CsgA^R62D/K107D^ and **(I)** Growth rates *k*_*2*_ of amyloid fiber propagation (linear scale) of CY^GK^ CsgA^R62D/K107D^. (Error bars represent SEM for ThT assay and SD for *k*_*1*_ and *k*_*2*_ of three replicates).

We next monitored aggregation kinetics at pH 4 and pH 5 in the presence of varying salt concentrations. At pH 4, with increasing salt concentrations, we observed no significant change to the nucleation and growth rates (Supplementary Figures S3A–I). The absence of negative charge on D78, D89, and D125 gatekeeper residues at pH 4 effectively abolished electrostatic interaction-based gatekeeping activity, leading to reduced effect of salt-mediated charge screening on nucleation and growth rates. The effect of salt did not significantly affect the nucleation rates of CY^GK^ CsgA^R62A/K107A^ and CY^GK^ CsgA^R62D/K107D^ at pH 5, however, the nucleation rate of CY^GK^ CsgA at pH 5 increased by more than 1 log between the lowest and highest salt concentration indicating some degree of electrostatic-mediated gatekeeping is occurring at the nucleation level (Supplementary Figures S4B, E, F). We further tested the electrostatic interactions between R62, K107 and D78, D89, and D125 gatekeeper residues by studying the aggregation kinetics of CY^GK^ CsgA, CY^GK^ CsgA^R62A/K107A^, and CY^GK^ CsgA^R62D/K107D^ at pH 6, which is above the theoretical pI of CY^GK^ CsgA. At pH 6, the equilibrium of the protonation state of D residues is predicted to shift more towards deprotonation compared to that at pH 5. We thus predicted that with increased negative charge on the D78, D89, and D125 residues, the gatekeeping function in CY^GK^ CsgA would be enhanced compared to that at pH 5, resulting in a longer lag phase and a larger effect from salt-mediated charge screening. At pH 6, in CY^GK^ CsgA we observed a three log decrease in the nucleation rates compared to at pH 5, while the nucleation rates of CY^GK^ CsgA^R62A/K107A^ decreased by only .6 log (Figures 3G, H). Interestingly, despite the disruption of gatekeeping function in CY^GK^ CsgA^R62D/K107D^, we observed 1.9 log lower nucleation rate at pH 6 compared to pH 5 (Figure 3H). The growth rates for the three proteins also displayed significant difference at pH 6 (Figure 3I; Table 1). In the presence of salt, the effect of gatekeeping activity in CY^GK^ CsgA at pH 6 was negatively affected suggesting that disruption of electrostatic interactions led to increased nucleation rates (Figures 4A, B). At the highest salt concentration of 600 mM, CY^GK^ CsgA nucleation rates were comparable to CY^GK^ CsgA nucleation rates at pH 4 and 5. Salt-mediated charge screening had no significant impact on the nucleation rates of CY^GK^ CsgA^R62A/K107A^ (Figures 4D, E), while in the case of CY^GK^ CsgA^R62D/K107D^ nucleation rates increased by two log with increasing salt concentration (Figures 4G, H). The growth rates showed a small increase for all the three proteins with approximately two times increase in the growth rates at 600 mM NaCl compared to 0 mM NaCl (Figures 4C, F, I). Increasing presence of negative charges on D residues led to stronger gatekeeping function specifically in CY^GK^ CsgA, however this charge was reversed by increasing salt-mediated charge screening.

**TABLE 1:** *T*-test for Nucleation Rate Constants and Autocatalytic Growth Constants at Various pH.

		pH 4		pH 5		pH 6	pH 7.3		pH 8
Nucleation rate constant *k*_*1*_ (*p*-value)									
CY^GK^ CsgA - CY^GK^ CsgA^R62A/K107A^		.079983		.035428		.172739	.000979		.021331
CY^GK^ CsgA - CY^GK^ CsgA^R62D/K107D^		.608020		.032945		.003791	.000018		.021406
CY^GK^ CsgA^R62A/K107A^ - CY^GK^ CsgA^R62D/K107D^		.588664		.788209		.183186	.002416		.006959
Autocatalytic growth constant *k*_*2*_ (*p*-value)									
CY^GK^ CsgA - CY^GK^ CsgA^R62A/K107A^		.009525		.029969		.042197	.058386		.000032
CY^GK^ CsgA - CY^GK^ CsgA^R62D/K107D^		.030473		.355798		.001302	.000001		.000045
CY^GK^ CsgA^R62A/K107A^ - CY^GK^ CsgA^R62D/K107D^		.228740		.010868		.013451	.000062		.000011
Nucleation Rate Constant *k*_*1*_ Between pH ranges (*p*-value)									
**Between pH:**	**pH 4 and 5**		**pH 5 and 6**		**pH 6 and 7.3**			**pH 7.3 and 8**	
CY^GK^ CsgA	.011172		.071501		.018492			.000059	
CY^GK^ CsgA^R62A/K107A^	.447643		.112145		.009265			.486193	
CY^GK^ CsgA^R62D/K107D^	.747897		.022939		.000021			.001047	

It is important to note that CY^GK^ CsgA contains other charged residues, including residues with p*K*_a_ values that falls within this pH range. Notably, all three proteins contain a 6-His tag as well as four native histidine residues. These residues would also trend towards deprotonation as the pH reaches and surpasses pH 6. It is possible that changes in the ionization state of other residues within CY^GK^ CsgA, CY^GK^ CsgA^R62A/K107A^, and CY^GK^ CsgA^R62D/K107D^ would also impact polymerization rates, however it does not appear that these changes account for the differences in the nucleation rate between CY^GK^ CsgA and the mutants CY^GK^ CsgA^R62A/K107A^ and CY^GK^ CsgA^R62D/K107D^.

### Diminished gatekeeping activity at pH 8 suggests deprotonation of positively charged residues

We next explored the aggregation kinetics of CY^GK^ CsgA, CY^GK^ CsgA^R62A/K107A^, and CY^GK^ CsgA^R62D/K107D^ at pH 8. The experimentally determined p*K*_a_ of K residues ranges from 6.5 to 12.12 while R residues have the highest p*K*_a_ among the ionizable groups and are thus rarely deprotonated at pH ≤ 10 (Isom et al., 2008; Harms et al., 2009; Harms et al., 2011; Pahari et al., 2019). At pH 8, buried K residues shift towards deprotonation, while other K and R residues likely remain protonated (Isom et al., 2011).

**FIGURE 5:**
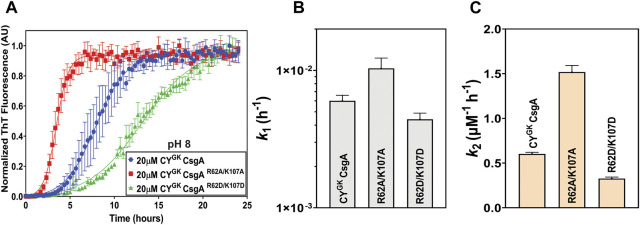
Loss of positive charge on K residues negatively impacts gatekeeping function. Analysis of aggregation kinetics of CY^GK^ CsgA, CY^GK^ CsgA^R62A/K107A^, and CY^GK^ CsgA^R62D/K107D^ at 37°C, pH 8. **(A)** ThT fluorescence assay. **(B)** Nucleation rates *k*_*1*_ of amyloidosis (log scale) and **(C)** Growth rates *k*_*2*_ of amyloid fiber propagation (linear scale). (Error bars represent SEM for ThT assay and SD for *k*_*1*_ and *k*_*2*_ of three replicates).

**FIGURE 6:**
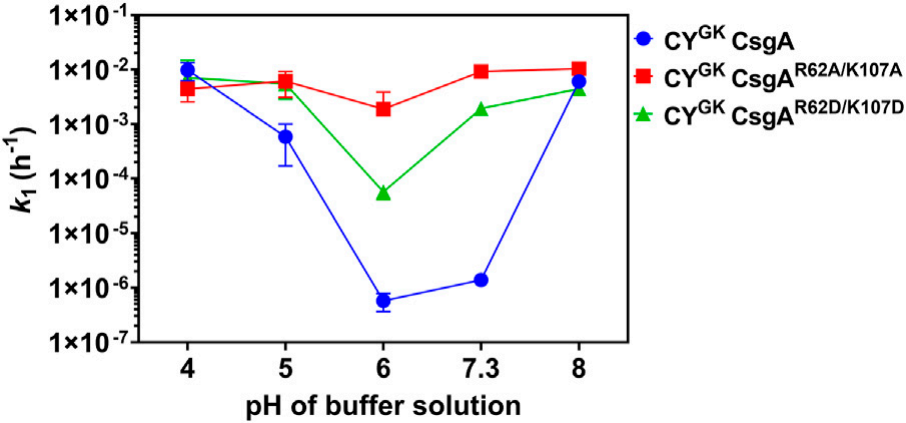
Comparison of the nucleation rates at different pH conditions. Nucleation rates k_1_ (log scale) of CY^GK^ CsgA (blue), CY^GK^ CsgA^R62A/K107A^ (red), and CY^GK^ CsgA^R62D/K107D^ (green) at different pH conditions at 37°C (Error bars represent SD for *k*_*1*_ of three replicates).

Interestingly, in CY^GK^ CsgA we observed 3.6 log increase in the nucleation rates compared to those at pH 7.3 with no significant difference in the nucleation rates of CY^GK^CsgA^R62A/K107A^ and CY^GK^ CsgA^R62D/K107D^ between pH 8 and pH 7.3 (Figure 1C, 5B). Overall, CY^GK^ CsgA, CY^GK^ CsgA^R62A/K107A^, and CY^GK^ CsgA^R62D/K107D^ had comparable nucleation rates at pH 8, with less than .4 log difference between the three proteins (Figures 5A, B). Based on our observations we suggest that the K residues in CY^GK^ CsgA are likely to be deprotonated at pH 8 and hence gatekeeping function would be negatively impacted. While K residues buried in the interiors of globular proteins have been shown to have significantly altered pKa values (Isom et al., 2011), in context of CsgA, which is an intrinsically disordered protein, our observations suggest that the impact of pH on the charged state of K residues might extend beyond the conformational state of the protein under study. Our observations were further supported by salt-mediated charge screening that showed less effect on the nucleation rates of CY^GK^ CsgA and CY^GK^ CsgA^R62A/K107A^ with less than .6 log difference between 0 mM and 600 mM NaCl (Supplementary Figures S5A, D). The nucleation rates of CY^GK^ CsgA^R62D/K107D^ showed one log increase at 600 mM NaCl compared to 0 mM NaCl (Supplementary Figure S5G). Interestingly, unlike the nucleation rates, the growth rates of CY^GK^ CsgA, CY^GK^ CsgA^R62A/K107A^, and CY^GK^ CsgA^R62D/K107D^ at pH 8 increased significantly with increasing salt concentrations (Supplementary Figures S5C, F, I) suggesting the involvement of other amino acid residues in amyloid fiber elongation. Trends in nucleation rate across the tested pH range for the CsgA variants are summarized in Figure 6.

### CY^GK^ CsgA, CY^GK^ CsgA^R62A/K107A^, and CY^GK^ CsgA^R62D/K107D^ mutants form curli fibers *in vivo*

During curli biogenesis, *E. coli* CsgA monomers are secreted to the extracellular space and the *in vivo* polymerization of CsgA is directed by a membrane associated CsgB nucleator protein *via* the Type VIII secretion system (Sujeet et al., 2019). CsgA homologs belonging to diverse species have been shown to be nucleated by *E. coli* CsgB both *in vitro* and *in vivo* (Zhou et al., 2013). In addition, wild-type CY CsgA can complement an *E. coli* Δ*csgA* strain (Zhou et al., 2013; Bhoite et al., 2022). To ensure that the addition of gatekeeper residues in CY CsgA did not compromise curli fiber formation, we tested polymerization of the CY CsgA variants *in vivo*. *E. coli* cells lacking endogenous CsgA were transformed with plasmids that expressed either an empty vector, EC wild-type CsgA, CY wild-type CsgA, CY^GK^ CsgA, CY^GK^ CsgA^R62A/K107A^, or CY^GK^ CsgA^R62D/K107D^ under the native *E. coli csgBAC* promoter in an *E. coli* MC 4100 Δ*csgA* strain. The N-terminal 22 amino acid sequence of *E. coli* CsgA (N22) was fused to all CsgA variants to facilitate extracellular export and assembly (Robinson et al., 2006; Hammer et al., 2012). The assembly of extracellular amyloid fibers was assessed by growing the cells on Congo red indicator plates. Strains that assemble extracellular cell surface associated fibers stain red on Congo red plates while the strains that cannot make extracellular cell surface associated fibers appear white or light pink (Zhou et al., 2013).

**FIGURE 7:**
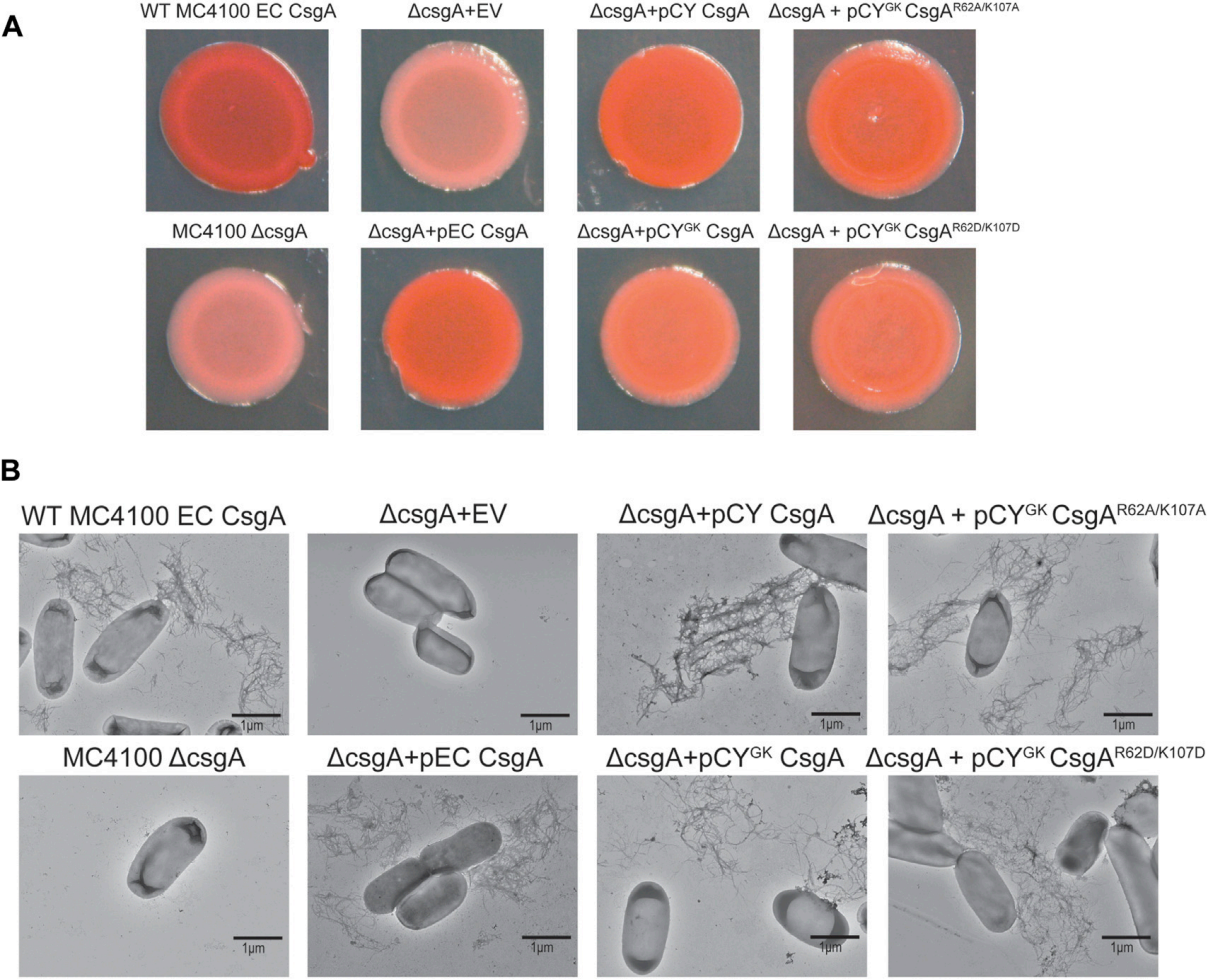
Complementation assay of CY^GK^ CsgA bacterial mutants. **(A)** *E. coli* MC4100 Δ*csgA* cells transformed with plasmids encoding the different CsgA mutants under the native *E. coli csgBAC* promoter and fused to *E. coli* CsgA sec signal sequence spotted on YESCA-Congo red indicator plates incubated for 48 h at 26°C (EV, empty vector). **(B)** Representative negative stain transmission electron micrographs of wild-type *E. coli* MC4100, *E. coli* MC4100 Δ*csgA*, *E. coli* MC4100 Δ*csgA* + EV*, E. coli* MC4100 Δ*csgA* + pEC CsgA, *E. coli* MC4100 Δ*csgA* + pCY CsgA, *E. coli* MC4100 Δ*csgA* + pCY^GK^ CsgA, *E. coli* MC4100 Δ*csgA* + pCY^GK^ CsgA^R62A/K107A^ and, *E. coli* MC4100 Δ*csgA* + pCY^GK^ CsgA^R62D/K107D^ (Scale bars, 1 µm).

Wild-type *E. coli* MC 4100 and the Δ*csgA* mutant strains with a plasmid expressing *E. coli* CsgA or wild-type CY CsgA formed red colonies after 48 h incubation indicating proper surface-anchored curli amyloid formation (Figure 7A). Light pink colonies were observed for the Δ*csgA* mutant strain and the Δ*csgA* mutant that contained the empty vector (Figure 7A). Interestingly, the Δ*csgA* mutant strains which contained plasmids that expressed either CY^GK^ CsgA, CY^GK^ CsgA^R62A/K107A^ or CY^GK^ CsgA^R62D/K107D^ formed red colored colonies, indicating that these mutants successfully secrete and assemble curli *in vivo* (Figure 7A). Whole-cell transmission electron microscopy (TEM) revealed the presence of cell-surface associated curli amyloid fibers in WILD-TYPE MC 4100, the Δ*csgA* mutant strain harboring an *E. coli* CsgA plasmid, and the Δ*csgA* mutant strain with a CY wild-type CsgA plasmid (Figure 7B). No cell surface-associated curli fibers were seen in the Δ*csgA* mutant strain and the Δ*csgA* mutant strain harboring the empty vector (Figure 7B). Cell-surface associated curli fibers were present in cells from the Δ*csgA* mutant strains harboring CY^GK^ CsgA, CY^GK^ CsgA^R62A/K107A^, and CY^GK^ CsgA^R62D/K107D^ encoding plasmids, and appeared to have similar morphology to fibers produced by Δ*csgA* with a CY wild-type CsgA plasmid (Figure 7B). Curli fibers were found on fewer cells in CY^GK^ CsgA, CY^GK^ CsgA^R62A/K107A^, and CY^GK^ CsgA^R62D/K107D^ mutants than on wild-type MC 4100 and the Δ*csgA* mutant strain with an *E. coli* CsgA expressing plasmid. Congo red staining was not apparent in the underlaying agar beneath the biofilms grown in Figure 7A indicating that fibers were not polymerizing without anchoring to the cell surface. However, non-surface anchored curli fibers were observed when imaging the CY^GK^ CsgA^R62A/K107A^ and CY^GK^ CsgA^R62D/K107D^ mutants.

## Discussion

### R62 and K170 residues are implicated in gatekeeping function

Sequence alignment of CsgA homologs revealed the presence of conserved R and K residues in addition to gatekeeper D residues previously found in *E. coli* CsgA (Supplementary Figure S1) (Wang et al., 2010). Charged residues like R, K, and D have been shown to function as gatekeeper residues in many proteins (Wang and Chapman, 2008; Reumers et al., 2009; Beerten et al., 2012). We thus hypothesized that the R and K residues in CsgA are also important for gatekeeping function. CY^GK^ CsgA is a variant of wild-type *C. youngae* CsgA and includes added D78, D89, and D125 gatekeeper residues. CY^GK^ CsgA natively contains the conserved R and K residues. These residues were replaced with alanine (CY^GK^ CsgA^R62A/K107A^) or aspartic acid (CY^GK^ CsgA^R62D/K107D^) to test whether these residues function as gatekeepers. This substitution resulted in a significant decrease in the lag phase and increase in the nucleation rates compared to CY^GK^ CsgA at pH 7.3 (Figures 1B, C, 6). This increase in the nucleation rates of CY^GK^ CsgA^R62A/K107A^ and CY^GK^ CsgA^R62D/K107D^ suggested that in addition to the D78, D89, and D125 gatekeeper residues, the R62 and K170 residues are implicated in gatekeeping function. The growth rates were not significantly affected in CY^GK^ CsgA or CY^GK^ CsgA^R62A/K107A^, but CY^GK^ CsgA^R62D/K107D^ showed 2 times reduction in growth rates (Figure 1D). This decrease in the growth rate could be attributed to the increased negative charge on the protein with the addition of gatekeeper D residues in the CY^GK^ CsgA background as well as the replacement of native positively charged lysine and arginine with aspartic acid. Charge repulsion has been shown to negatively impact amyloidogenesis (Guo et al., 2005; Sahoo et al., 2009; Shammas et al., 2011). The repulsion between negatively charged aspartic acid (D) residues likely acts as a gatekeeper at the growth phase due to the accumulation of negative charge in the growing CY^GK^ CsgA^R62D/K107D^ fiber.

### Disrupting electrostatic interaction between gatekeeper residues reduces gatekeeping activity

At pH 7.3, R and K residues are predominantly positively charged while D residues are predominantly negatively charged. As increased nucleation rates were observed when R62 and K107 residues were substituted to charge insensitive A or negatively charged D residues (at pH 7.3) (Figure 1C), we hypothesized that the electrostatic interactions between R62, K107 and D78, D89, and D125 gatekeeper residues were responsible for gatekeeping function. We used salt-mediated charge screening to neutralize the charge on R62, K107, D78, D89, and D125 residues. In CY^GK^ CsgA, the charge screening had the highest impact on aggregation kinetics and nucleation rate compared to CY^GK^ CsgA^R62A/K107A^ and CY^GK^ CsgA^R62D/K107D^ (Figures 2A, B, D, E, G, H) which would not have R-D or K-D electrostatic interactions acting as gatekeepers. The significantly higher nucleation rate in CY^GK^ CsgA in the presence of salt suggested that the salt-mediated charge screening of the R, K, and D residues prevented electrostatic interactions between them resulting in disruption of gatekeeping activity that is acting at the nucleation level. In the case of CY^GK^ CsgA^R62A/K107A^, and CY^GK^ CsgA^R62D/K107D^, the absence of R62 and K107 residues made their nucleation rates less sensitive to salt-mediated charge screening. To further substantiate our claim that electrostatic interactions are gatekeeping CsgA polymerization, we measured the aggregation kinetics at various pH conditions. At pH 4, the equilibrium of protonation state of D residues shifted more towards protonation. In the absence of negative charge, D78, D89, and D125 residues no longer functioned as gatekeepers which was reflected in a decreased lag phase and increased nucleation rate of CY^GK^ CsgA (Figures 3A, B). In CY^GK^ CsgA^R62A/K107A^ and CY^GK^ CsgA^R62D/K107D^, the absence of positively charged R62 and K107 residues in addition to the lack of negative charge on D78, D89, and D125 gatekeeper residues abolished the gatekeeping function (Figures 3A, B). At pH 5, CY^GK^ CsgA displayed a longer lag phase of aggregation and 1.2 log lower nucleation rate than at pH 4 while the nucleation rates of CY^GK^ CsgA^R62A/K107A^ and CY^GK^ CsgA^R62D/K107D^ were not affected (Figures 3D, E). At pH 5, as the solution pH surpassed the p*K*_a_ of carboxylic group of D residues, the equilibrium of protonation state of D residues shifted more towards deprotonation and hence the net negative charge on D residues increased (Blaber, 2019). This increase in the negative charge on D residues resulted in increased gatekeeping activity in CY^GK^ CsgA (Figures 3D, E). Despite the increased negative charge on D78, D89, and D125 residues, due to the lack of positively charged R62 and K107 residues in CY^GK^ CsgA^R62A/K107A^ and CY^GK^ CsgA^R62D/K107D^, the nucleation rates at pH 5 were not affected (Figure 3E). These observations suggested that a net negative charge on D residues was necessary for gatekeeping activity *via* electrostatic interactions between R62, K107 and D78, D89, and D125. Comparison of CY^GK^ CsgA and CY^GK^ CsgA^R62A/K107A^ nucleation rate constant *k*_1_ yielded a p-value indicating a non-significant change (Table 1). This value is due to the standard deviation between replicates. As can be seen in Figure 6, nucleation rate *k*_1_ had the most drastic change between pH 5 and 6, especially when comparing CY^GK^ CsgA and CY^GK^ CsgA^R62A/K107A^.

As the solution pH surpassed the pI of CY^GK^ CsgA, the degree of deprotonation of D residues increased. With increased negative charge on D residues in CY^GK^ CsgA we observed the lowest nucleation rates at pH 6 compared to that at any other pH (Figure 6). Interestingly, at pH 6 due to the absence of R62 and K107 residues in CY^GK^ CsgA^R62A/K107A^, the nucleation rates did not show any significant decrease while CY^GK^ CsgA^R62D/K107D^ showed 1.9 log reduction in nucleation rates compared to pH 5 (Figure 3H). The substitution of R62 and K107 residues with D residues in CY^GK^ CsgA^R62D/K107D^ along with the increased negative charge on D residues at pH 6 resulted in charge repulsion. This could explain the delayed nucleation and slower growth as has been reported in other amyloid proteins (Wang et al., 2010; Beerten et al., 2012). Moreover, increasing salt concentration and charge screening led to increased nucleation and growth rates further supporting the idea of charge repulsion in absence of salt leading to delayed nucleation and growth rates in CY^GK^ CsgA^R62D/K107D^ at pH 6 (Figures 4G–I). Similarly, increasing salt concentrations at pH 6 led to increased nucleation and growth rates in CY^GK^ CsgA due to screening of the negative charge on D residues (Figures 4A–C). The nucleation and growth rates of CY^GK^ CsgA^R62A/K107A^ were not affected due to the absence of R62 and K107 mediated electrostatic interaction with D residues (Figures 4D–F).

The increased presence of negative charge on D residues led to greater gatekeeping activity. We then explored the effect of charge neutralization of the positively charged R62 and K107 residues on gatekeeping function. At pH 8, we have shown that K residues are likely partially deprotonated in CY^GK^ CsgA. While the D residues were negatively charged, the partial deprotonation of K residues at pH 8 negatively impacted the gatekeeping activity (Figures 5A, B). The loss of electrostatic interactions between the D and K residues resulted in increased nucleation rates in CY^GK^ CsgA compared to those at pH 7.3 (Figures 5A–C). The lack of electrostatic interaction due to the absence of R62 and K107 residues was further demonstrated in the similar nucleation rates of CY^GK^ CsgA^R62A/K107A^ and CY^GK^ CsgA^R62D/K107D^ at pH 7.3 (Figure 6 and compare Figure 1C, 5B). As the electrostatic interactions were disrupted due to the deprotonation (CY^GK^ CsgA) or absence of K107 residue (CY^GK^ CsgA^R62A/K107A^), salt-mediated charge screening did not show any significant increase in the nucleation rates (Supplementary Figures S5B, E). For CY^GK^ CsgA^R62D/K107D^, the 1 log increase in the nucleation rates at the highest salt concentration could be attributed to the screening of negative charges on D residues as charge repulsion has been shown to slow down amyloid fiber growth (Supplementary Figure S5H) (Abdolvahabi et al., 2015; Vettore and Buell, 2019).

### CY^GK^ CsgA, CY^GK^ CsgA^R62A/K107A^, and CY^GK^ CsgA^R62D/K107D^ can complement Δ*csgA E. coli*

To ensure that mutations to the R and K residues did not significantly affect curli fiber formation, we expressed CY^GK^ CsgA, CY^GK^ CsgA^R62A/K107A^, and CY^GK^ CsgA^R62D/K107D^ in *E. coli* cells lacking CsgA. Our earlier studies have shown that CsgA homologs from diverse species could complement *E. coli* CsgA deletion *in vivo* (Zhou et al., 2012). CY^GK^ CsgA, CY^GK^ CsgA^R62A/K107A^, and CY^GK^ CsgA^R62D/K107D^ successfully complemented *E. coli* CsgA deletion *in vivo* as seen by the red colored colonies on Congo red indicator plates (Figure 7A). Whole-cell analysis confirmed the presence of surface-associated curli fibers in *E. coli* Δ*csgA* strains which harbored plasmids expressing CY^GK^ CsgA, CY^GK^ CsgA^R62A/K107A^, or CY^GK^ CsgA^R62D/K107D^ (Figure 7B). No obvious morphological differences were observed in the assembled curli fibers of cells expressing wild-type CY CsgA, CY^GK^ CsgA, CY^GK^ CsgA^R62A/K107A^, or CY^GK^ CsgA^R62D/K107D^ indicating that the addition of gatekeeper residues did not significantly affect fiber assembly. Few CY^GK^ CsgA, CY^GK^ CsgA^R62A/K107A^, and CY^GK^ CsgA^R62D/K107D^ cells presented with surface-anchored curli fibers while most cells from *E. coli* Δ*csgA* with plasmids expressing EC wild-type CsgA or CY wild-type CsgA had surface-anchored curli fibers. Congo red staining induced by the formation of non-surface associated curli fibers was not observed in the agar underlying the CY^GK^ CsgA^R62A/K107A^ and CY^GK^ CsgA^R62D/K107D^ biofilms, indicating that non-surface attached fibers observed by TEM likely detached during grid preparation or staining. As these variants have introduced D residues, as well as mutations to the positively charged lysine and arginine residues, it is possible that fiber stability is compromised due to accumulation of negative charge which may be exacerbated by the addition of negatively charged staining agent uranyl acetate.

In this report, in addition to the earlier characterized D78, D89, and D125 residues, we identify R62 and K107 residues as new gatekeeper residues in bacterial functional amyloid CY^GK^ CsgA. We provide preliminary evidence behind the mechanism by which R62, K107, and D78, D89, and D125 residues function as gatekeepers in CY^GK^ CsgA. The resulting electrostatic interactions between these oppositely charged residues allows for the formation of a stable contact slowing down formation of an amyloid-competent pre-fibrillar structure, thereby modulating amyloidogenesis. As this interaction occurs prior to CsgA monomers forming the characteristic β-sheet formation required for nucleation, we cannot visualize these gatekeeper interactions in predicted structures illustrating fully folded CY CsgA. Our *in vitro* study would benefit from additional studies using HDX-mass spectrometry, NMR etc. To shed light on the role of electrostatic interactions in the formation and structure of bacterial functional amyloids. Our study thus lays the foundation for understanding an electrostatic interaction-based biochemical mechanism that controls CY^GK^ CsgA nucleation.

## Materials and methods

### Protein purification

CY^GK^ CsgA, CY^GK^ CsgA^R62A/K107A^, and CY^GK^ CsgA^R62D/K107D^ CsgA were purified with certain modifications (Zhou et al., 2013). Briefly, cell pellets of CY^GK^ CsgA, CY^GK^ CsgA^R62A/K107A^, and CY^GK^ CsgA^R62D/K107D^ were first treated with 2 mL 1,1,1,3,3,3-hexafluoro-2-propanol (HFIP) and incubated at room temperature (RT) for 10 min with intermittent mixing. Following this, 25 mL of 8 M guanidine hydrochloride in 50 mM of potassium phosphate buffer (KPi) pH 7.3 was added to the cell lysate and incubated on a rocker for 1 h at RT. The solution was then centrifuged at 10,000 g for 20 min at 4°C. The supernatant was collected and sonicated three times for 20 s each. 800 µL of Sigma HIS-Select^®^ HF Nickel Affinity Gel beads were added to the sonicated supernatant and incubated on a rocker for 1 h at RT. The protein was eluted with 125 mM imidazole in 50 mM KPi pH 7.3 (Zhou et al., 2013). Following elution, the proteins were buffer exchanged to the buffer pH of choice using Thermo Scientific Zeba™ Spin Desalting Columns 7k MWCO. The protein concentration after buffer exchange was assayed using Thermo Scientific Pierce™ Rapid Gold BCA Protein Assay Kit. Primers used to make mutant strains are listed in Table 2 and strains are listed in Table 3.

**TABLE 2:** List of oligonucleotide primers used.

Description	Sequence	Purpose
105_C127G_G128C	5′-TGC TCT GCA AAG CGA TGC GGC TAA ATC AGA TGT CAC TAT C-3′	Site directed mutagenesis primers to mutate the positive K and R in CY^GK^ CsgA to Alanine (A)
106_A262G_A263C	5′-ACT ATC GAT CAG TGG AAT GCG GCA AAT GCT GAT ATT AGC GTG AC-3′	
110_C128A	5′- CTC TGC AAA GCG ATG CGG ATA AAT CAG ATG TCA CTA T -3′	Site directed mutagenesis primers to mutate the Alanine in CY^GK^ CsgA^R62A/K107A^ to Aspartic Acid (D)
111_C263A_A264T	5′- TAT CGA TCA GTG GAA TGC GGA TAA TGC TGA TAT TAG CGT GAC -3′	
77_pLR5_CY_Fow	5′-TAA CCA ACA CTA AGG ATC CTC TAG AGT CGA C-3′	To linearize pLR5 with CY^GK^ CsgA overhangs for Gibson assembly
78_pLR5_CY_Rev	5′-AGC GAA GAA TTT GGG CCG CTA TTA TTA CC-3′	
79_CYf_pLR5_Fow	5′-GCC CAA ATT CTT CGC TGA GTA TCT ACC AAT ATG G-3′	To amplify CY^GK^ CsgA with pLR5 overhangs for Gibson assembly
80_CYf_pLR5_Rev	5′-AGG ATC CTT AGT GTT GGT TAG CTG TTG CAT TG-3′	

**TABLE 3:** Strains and plasmids used in this study.

	Relevant characteristics	References
Strains		
LSR10	MC4100 *ΔcsgA*	Yun et al. (2007)
NEB 3016	T7 Express *I* ^*q*^ Competent *E. coli*	NEB Inc.
Plasmids		
pLR2	Control vector containing *E. coli csgBA* promoter	Yun et al. (2007)
pLR5	*E. coli csgA* sequence cloned in pLR2	Hammer et al. (2007)
pLR2_CY WT CsgA	CY wild-type CsgA sequence cloned in pLR2	Bhoite et al. (2022)
pLR2_CY^GK^ CsgA	CY^GK^ CsgA sequence cloned in pLR2	Bhoite et al. (2022)
pLR2_CY^GK^ CsgA^R62A/K107A^	CY^GK^ CsgA^R62A/K107A^ sequence cloned in pLR2	This study
pLR2_CY^GK^ CsgA^R62D/K107D^	CY^GK^ CsgA^R62D/K107D^ sequence cloned in pLR2	This study
pET28a	IPTG inducible expression vector	NEB Inc.
pET28a_CY^GK^ CsgA	C-terminal His6 tagged CY^GK^ CsgA cloned in pET28a	Bhoite et al. (2022)
pET28a_CY^GK^ CsgA^R62A/K107A^	C-terminal His6 tagged CY^GK^ CsgA^R62A/K107A^ cloned in pET28a	This study
pET28a_CY^GK^ CsgA^R62D/K107D^	C-terminal His6 tagged CY^GK^ CsgA^R62D/K107D^ cloned in pET28a	This study

### Thioflavin-T assay

The aggregation kinetics of CY^GK^ CsgA, CY^GK^ CsgA^R62A/K107A^ or CY^GK^ CsgA^R62D/K107D^ CsgA was monitored in black flat-bottom 96-well plates using fluorescent dye Thioflavin-T (ThT) in an automated microtiter plate reader (Tecan Infinite M200). Freshly purified CY^GK^ CsgA, CY^GK^ CsgA^R62A/K107A^ or CY^GK^ CsgA^R62D/K107D^ CsgA was diluted to a final concentration of 20 µM in presence or absence of varying concentrations of NaCl. The samples were incubated at 37°C under quiescent conditions in presence of 20 µM ThT. The ThT fluorescence intensity was recorded every 20 min with orbital shaking for 5 s before the readings (excitation: 438 nm; emission: 495 nm). All experiments were performed in triplicates with at-least three biological replicates and nucleation and growth rates were calculated using the following equation (Morris et al., 2008).

$$Y=a-\frac{\left(\frac{k_{1}}{k_{2}}\right)+a}{1+\left(\left(\frac{k_{1}}{k_{2}*a}\right)*\mathrm{EXP}\left(\left(k_{1}+k_{2}*a\right)*x\right)\right)}$$

Where a is the final value of Y at the end of the reaction, $k_{1}$ is the nucleation rate and $k_{2}$ is the growth rate. ThT assays comparing different CsgA mutants were conducted separately.

### Complementation assay

Overnight cultures in LB broth at 37°C of wild-type *E. coli* MC 4100 or wild-type *E. coli* MC 4100 Δ*csgA* cells expressing either empty vector (EV), *E. coli* CsgA, CY CsgA, CY^GK^ CsgA, CY^GK^ CsgA^R62A/K107A^ or CY^GK^ CsgA^R62D/K107D^ CsgA were pelleted and diluted to 1.0 OD_600nm_ in YESCA (yeast extract, casamino acids). 4 µL was spotted on YESCA agar plates supplemented with 50 µg/mL Congo red and incubated at 26°C for 48 h to induce CsgA expression. Images were recorded using Canon EOS Rebel XSi camera and the background Congo red color was edited out in Adobe Photoshop.

### Transmission electron microscopy (TEM)

For whole-cell imaging, wild-type *E. coli* MC 4100 or *E. coli* MC 4100 Δ*csgA* cells expressing either empty vector (EV), *E. coli* CsgA, CY CsgA, CY^GK^ CsgA, CY^GK^ CsgA^R62A/K107A^ or CY^GK^ CsgA^R62D/K107D^ CsgA were grown on YESCA-agar plates supplemented with Congo red for 48 h at 26°C. After incubation, the cells were scraped from the YESCA-agar plates and re-suspended to 1.0 OD_600nm_ in 50 mM potassium phosphate buffer pH 7.3 before applying 5 µL of the cell suspension to formvar-coated grids followed by staining with 1% uranyl acetate solution. Samples were imaged on Jeol electron microscope (JEOL1400plus).

## Data Availability

The original contributions presented in the study are included in the article/Supplementary Material, further inquiries can be directed to the corresponding author.
