# Supplementary material for: Electrostatic interactions mediate the nucleation and growth of a bacterial functional amyloid
Source: Front Mol Biosci. 2023 Jan 12;10:1070521. doi: 10.3389/fmolb.2023.1070521 (PMC9900396; doi:10.3389/fmolb.2023.1070521)
Supplement: Supplementary file 1 [file DataSheet1.PDF]

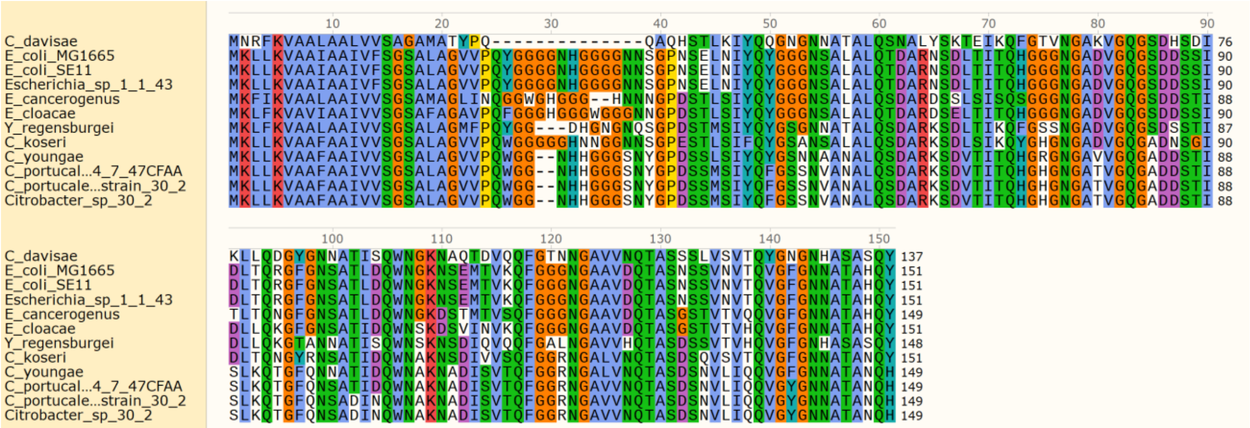

Figure S1. Sequence alignment of various CsgA homologs. The positively charged arginine (R) and lysine (K) residues show greater degree of conservation than the negatively charged aspartic acid (D) residues.

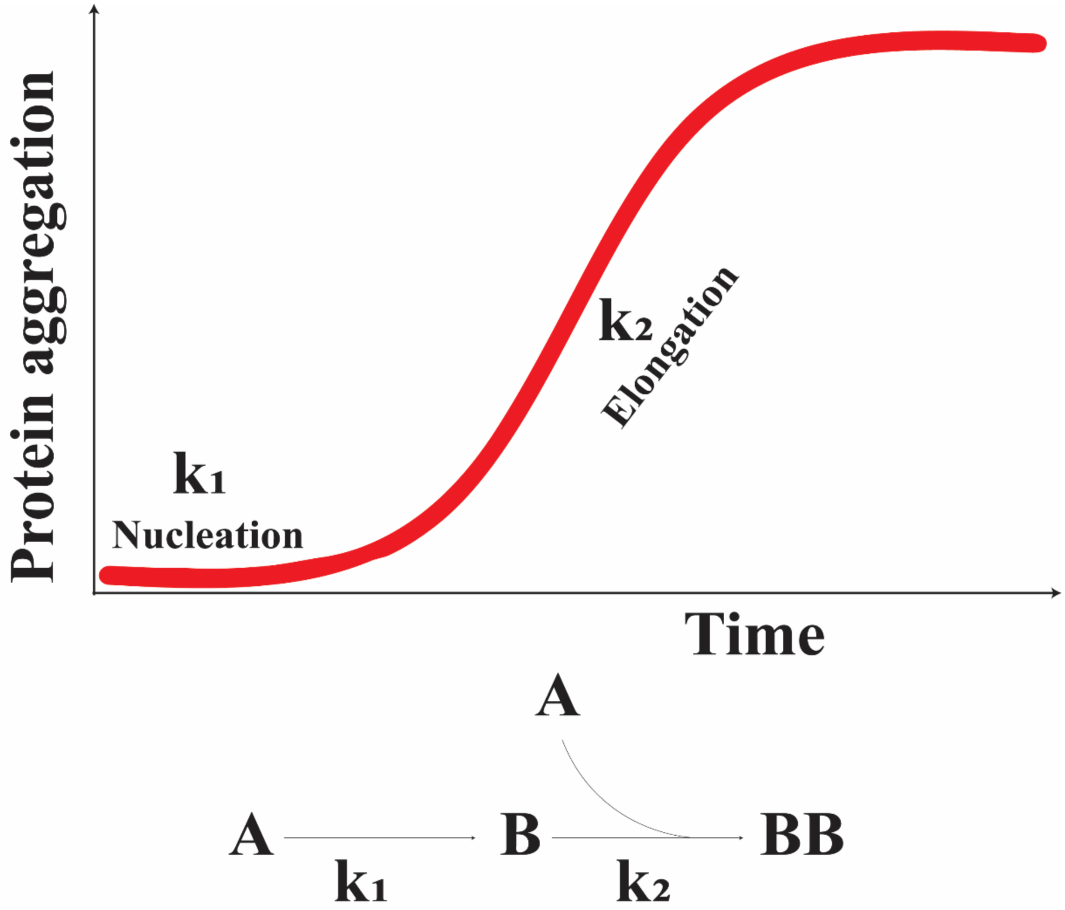

Figure S2. Schematics of the nucleation k1 and elongation k2 phase of amyloidogenesis. The protein monomers ‘A’ form nuclei “B” with rate constant k1, followed by fast autocatalytic surface growth by addition of monomeric “A” with rate constant k2.

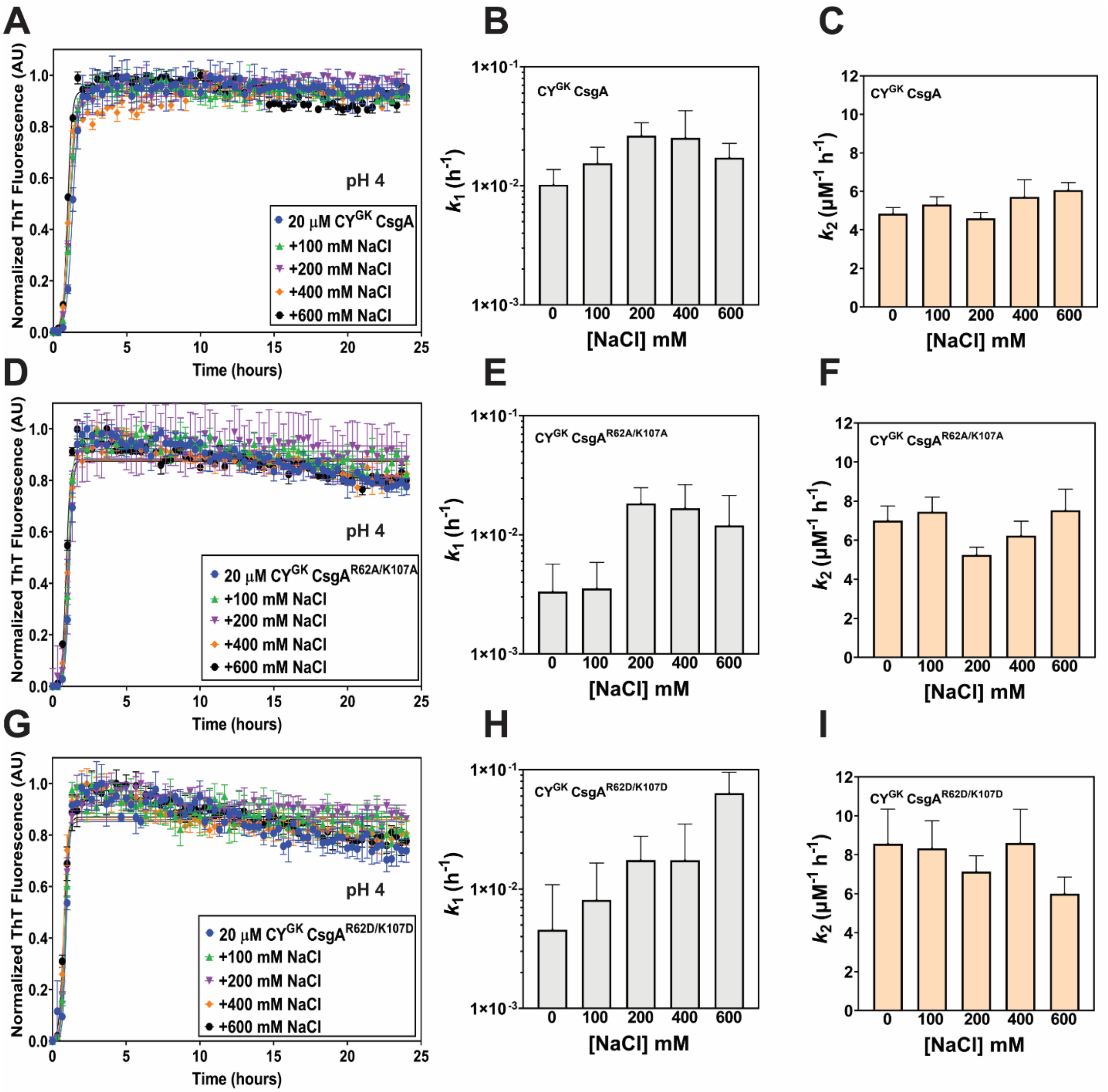

Figure S3. Salt has minimal impact on the aggregation kinetics at pH 4. Analysis of aggregation kinetics of CY^{GK} CsgA, CY^{GK} CsgA$^{R62A/K107A}$, and CY^{GK} CsgA$^{R62D/K107D}$ at 37°C, pH 4 in presence of increasing NaCl concentrations. **(A)** ThT fluorescence assay of CY^{GK} CsgA. **(B)** Nucleation rates k_1 of amyloidosis (log scale) of CY^{GK} CsgA. **(C)** Growth rates k_2 of amyloid fiber propagation (linear scale) of CY^{GK} CsgA. **(D)** ThT fluorescence assay of CY^{GK} CsgA$^{R62A/K107A}$. **(E)** Nucleation rates k_1 of amyloidosis (log scale) of CY^{GK} CsgA$^{R62A/K107A}$. **(F)** Growth rates k_2 of amyloid fiber propagation (linear scale) of CY^{GK} CsgA$^{R62A/K107A}$. **(G)** ThT fluorescence assay of CY^{GK} CsgA$^{R62D/K107D}$. **(H)** Nucleation rates k_1 of amyloidosis (log scale) of CY^{GK} CsgA$^{R62D/K107D}$ and **(I)** Growth rates k_2 of amyloid fiber propagation (linear scale) of CY^{GK} CsgA$^{R62D/K107D}$. (Error bars represent SEM for ThT assay and SD for k_1 and k_2 of three replicates).

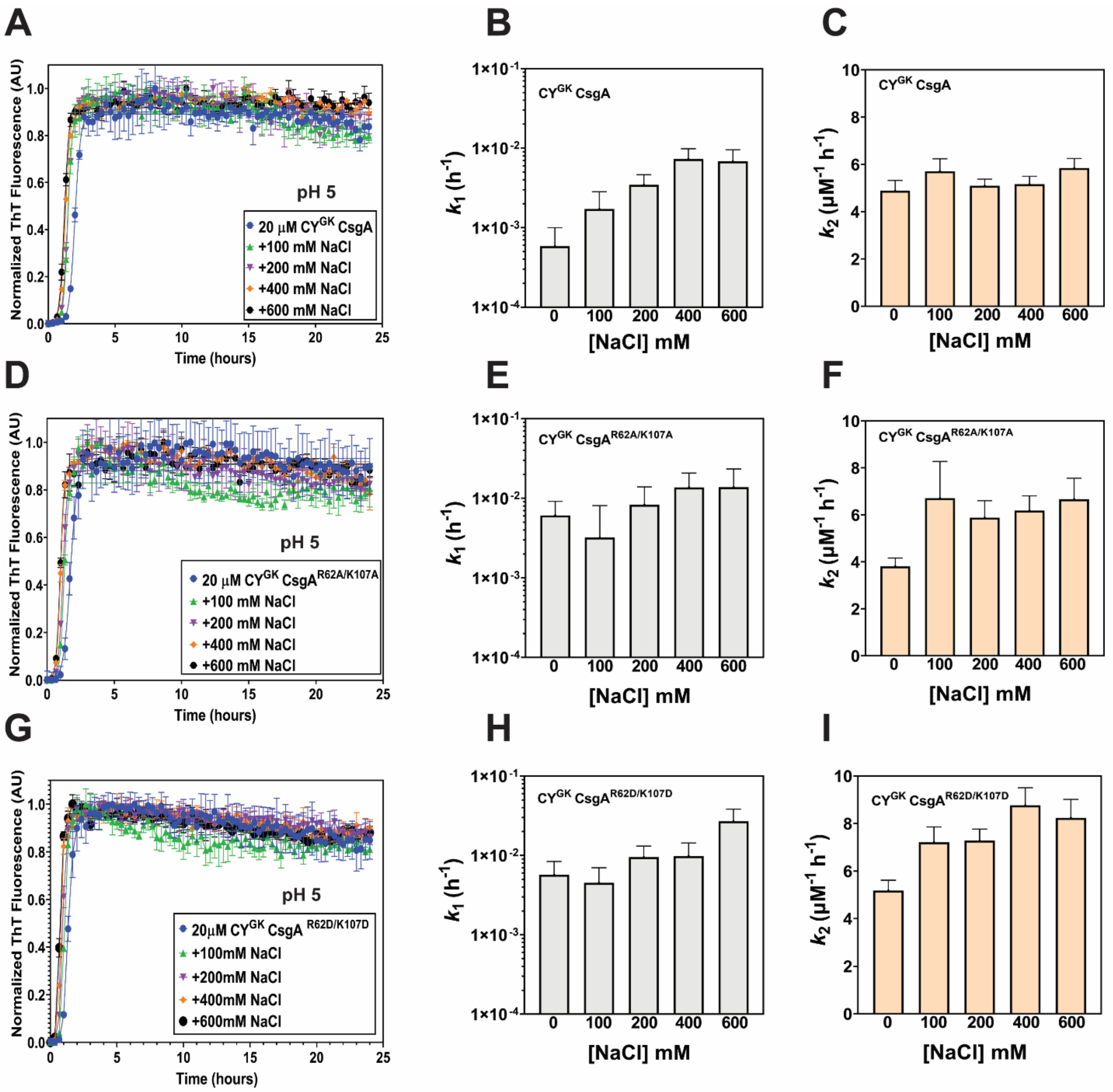

Figure S4. Salt-mediated charge screening of D residues at pH 5 accelerates aggregation. Analysis of aggregation kinetics of CY^{GK} CsgA, CY^{GK} CsgA$^{R62A/K107A}$, and CY^{GK} CsgA$^{R62D/K107D}$ at 37°C, pH 5 in presence of increasing NaCl concentrations. **(A)** ThT fluorescence assay of CY^{GK} CsgA. **(B)** Nucleation rates k_1 of amyloidosis (log scale) of CY^{GK} CsgA. **(C)** Growth rates k_2 of amyloid fiber propagation (linear scale) of CY^{GK} CsgA. **(D)** ThT fluorescence assay of CY^{GK} CsgA$^{R62A/K107A}$. **(E)** Nucleation rates k_1 of amyloidosis (log scale) of CY^{GK} CsgA$^{R62A/K107A}$. **(F)** Growth rates k_2 of amyloid fiber propagation (linear scale) of CY^{GK} CsgA$^{R62A/K107A}$. **(G)** ThT fluorescence assay of CY^{GK} CsgA$^{R62D/K107D}$. **(H)** Nucleation rates k_1 of amyloidosis (log scale) of CY^{GK} CsgA$^{R62D/K107D}$ and **(I)** Growth rates k_2 of amyloid fiber propagation (linear scale) of CY^{GK} CsgA$^{R62D/K107D}$. (Error bars represent SEM for ThT assay and SD for k_1 and k_2 of three replicates).

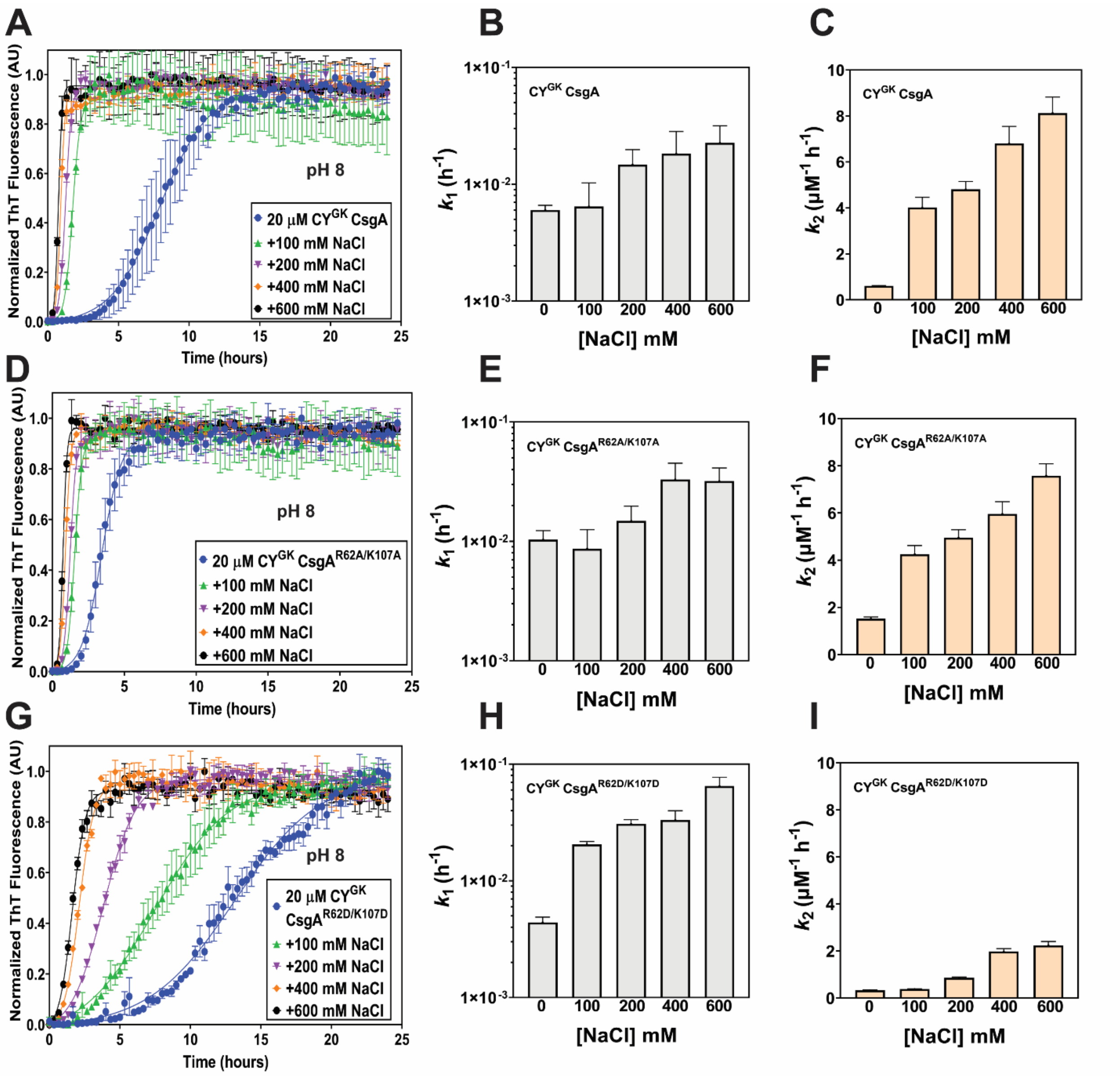

Figure S5. Effect of salt on the aggregation kinetics at pH 8.
Analysis of aggregation kinetics of CY^{GK} CsgA, CY^{GK} CsgA$^{R62A/K107A}$, and CY^{GK} CsgA$^{R62D/K107D}$ at 37°C, pH 8 in presence of increasing NaCl concentrations. **(A)** ThT fluorescence assay of CY^{GK} CsgA. **(B)** Nucleation rates k_1 of amyloidosis (log scale) of CY^{GK} CsgA. **(C)** Growth rates k_2 of amyloid fiber propagation (linear scale) of CY^{GK} CsgA. **(D)** ThT fluorescence assay of CY^{GK} CsgA$^{R62A/K107A}$. **(E)** Nucleation rates k_1 of amyloidosis (log scale) of CY^{GK} CsgA$^{R62A/K107A}$. **(F)** Growth rates k_2 of amyloid fiber propagation (linear scale) of CY^{GK} CsgA$^{R62A/K107A}$. **(G)** ThT fluorescence assay of CY^{GK} CsgA$^{R62D/K107D}$. **(H)** Nucleation rates k_1 of amyloidosis (log scale) of CY^{GK} CsgA$^{R62D/K107D}$ and **(I)** Growth rates k_2 of amyloid fiber propagation (linear scale) of CY^{GK} CsgA$^{R62D/K107D}$. (Error bars represent SEM for ThT assay and SD for k_1 and k_2 of three replicates).
